# Intraoperative optical coherence tomography assisted analysis of pars Plana vitrectomy for retinal detachment in morning glory syndrome: a case report

**DOI:** 10.1186/s12886-017-0533-0

**Published:** 2017-08-01

**Authors:** Lyubomyr M. Lytvynchuk, Carl G. Glittenberg, Siamak Ansari-Shahrezaei, Susanne Binder

**Affiliations:** 1 Department of Ophthalmology, Justus-Liebig-University Giessen, Eye Clinic, University Hospital Giessen and Marburg GmbH, Campus Giessen, Friedrichstrasse 18, 35392, Giessen, Germany; 2 Department of Ophthalmology, Rudolf Foundation Clinic, Juchgasse 25, A-1030 Vienna, Austria; 3 Karl Landsteiner Institute for Retinal Research and Imaging, Juchgasse 25, A-1030 Vienna, Austria; 4 Department of Ophthalmology, Medical University of Graz, Graz, Austria; 5 Retina Center Vienna, Jacquingasse 41, 1030 Vienna, Austria

**Keywords:** Morning glory syndrome, Non-rhegmatogenous retinal detachment, Intraoperative optical coherent tomography (iOCT), Pars plana vitrectomy, Epiretinal membrane

## Abstract

**Background:**

The pathogenesis of non-rhegmatogenous retinal detachment (non-RRD) associated with morning glory syndrome (MGS) is not established, as well as best surgical approach to treat RD. Our purpose was to analyse intraoperative optical coherence tomography data (iOCT) in all steps of pars plana vitrectomy (PPV) for non-RRD in MGS, in order to follow pathophysiological aspects of the disease and to understand the tissues behaviour during surgical workflow.

**Case presentation:**

Intraoperative spectral domain optical coherent tomography (iSD-OCT) assisted PPV using Rescan 700 (Carl Zeiss Meditech, Jena, Germany) with epiretinal membrane (ERM) and internal retinal membrane (ILM) peeling, and air endotamponade was performed on the only eye of a 21 years old female with non-RRD associated with MGS. BCVA, pre-, intra- and postoperative OCT were performed along with standard ocular examination. iOCT video and snapshots were analysed intra- and postoperatively using post-processing approach using graphic software. The progression of non-RRD resulted in best corrected visual acuity (BCVA) decrease from 0.8 to 0.2. Triamcinolone enhanced iOCT imaging revealed strong vitreous traction and adhesion above the macula and optic disc. Internal limiting membrane was peeled under iOCT control to prevent the peeling of inner layers of the retinal schisis. No retinal break was detected, and only air endotamponade was performed. The retina reattached during first 4 weeks of follow-up with gradual resolution of intraretinal- and subretinal fluid, and remained stable in 12 months. BCVA improved to 0.8.

**Conclusion:**

Based on iSD-OCT findings we assume that non-RRD in this case of MGS is caused primarily by the vitreous traction with further possible formation of the retinal breaks. Retinal reattachment reached only with air endotamponade strongly advocates the tractional component of non-RRD and retinal schisis assotiated with MGS. Early PPV for central non-RRD and retinal schisis with the use of iOCT can be performed in more safe and controlled manner and has to be considered to reduce the risk of retinal break formation and to prevent the central vision loss.

**Electronic supplementary material:**

The online version of this article (doi:10.1186/s12886-017-0533-0) contains supplementary material, which is available to authorized users.

## Background

Abnormalities of central retina, such as retinal detachment and retinal schisis can accompany congenital cavitary optic disc anomalies, to which belong optic disc coloboma, morning glory syndrome and extrapapillary cavitation. The morning glory optic disc abnormality or morning glory syndrome (MGS), that is also known as Handmann’sches Anomaly, is a congenital malformation of the intraocular and intrascleral parts of the optic nerve associated with a colobomatous defect of the optic nerve head [1–5]. Retinal detachment (RD) with retinal schisis are the most severe complications of MGS that impair visual acuity, with incidence 26–38% of patients with MGS [6]. Different pathogenesis theories of RD, including exudative, tractional and rhegmatogenous, were suggested [7–9], but non has enough of proofs. Up till now there are also no strict recommendation regarding type and timing of surgical intervention in order to treat RD, which usually involves macular area.

Intraoperative spectral-domain optical coherence tomography (iSD-OCT, iOCT) is an innovative diagnostic technique which already has obtained an important place in ophthalmic surgery of anterior and posterior segment of the eye. The use of iOCT systems has changed the understanding of pathogenesis of certain rare retinal disorders. It has been used during different vitreo-retinal surgeries bringing a remarkable assistance to perform the intervention in more safe manner and to reach the best postoperative results. The feasibility of iSD-OCT in eye surgery was previously assessed and reported [10–12]. Besides, iSD-OCT can serve to find out more about pathogenesis of rare posterior fundus disorders, due to live imaging and analysis of tissue behaviour during the surgery [28].

The aim of this study was to analyse all steps of pars plana vitrectomy for non-RRD in MGS using iSD-OCT data, in order to evaluate the use of a new imaging technique during surgery on the only eye with rare diseases and to better understand pathophysiological aspects and tissues behaviour during surgical workflow.

## Case presentation

### Methods

The study followed the tenets of the Declaration of Helsinki, informed consent was obtained from the patient after explanation of the nature and possible consequences of the study. The study was approved by the institutional Human Experimentation Committee at Rudolf Foundation Hospital (Vienna, Austria). The signed consent was obtained from the patient according to the Ethic Committee Regulations. Additionally, the consent form to use and publish examination data (including individual details, images and videos) was signed by the patients according to the Ethic Committee Regulations at Rudolf Foundation Hospital (Vienna, Austria)

### Patient

Twenty-one years old female presented with MGS on her only right eye (RE). The left eye was blind since childhood due to microphthamus aesthetically treated with an epiprosthesis. Best corrected visual acuity (BCVA) on the right eye was stable at 0.8 (ETDRS chart) during last 6 years starting from the first visit in our clinic. Then it slowly decreased to 0.2 (ETDRS chart) due to central non-RRD, retinal schisis and dense vitreous membranes.

### Ophthalmic examination

Best corrected visual acuity, non-contact tonography, visual fields, indirect slit-lamp funds ophthalmoscopy, ultrasound examination were performed on the RE pre-, postoperatively and in 1, 3, 6, 8 and 12 months of follow-up. Pre- and postoperative OCT scans of the macula and optic disc were performed using Cirrus™ HD-OCT 4000 (Carl Zeiss Meditech, Jena, Germany). For acquisition the Macular Cube 512 × 128 and the Optic Disc Cube 200 × 200 modes were used. OCT data were analysed using Macular Thickness Analysis, Advanced Visualisation and 3D visualisation.

### Surgery

Twenty-three gauge iSD-OCT assisted pars plana vitrectomy with epiretinal membrane (ERM) and internal limiting membrane (ILM) removal, and air endotamponade were considered on the patients’ RE and performed on 09.12.2014. The posterior vitreous was stained with triamcinolone acetonide (TA), and ERM and ILM were stained with Membraneblue Dual ® (D.O.R.C. International B.V., Zuidland, The Netherlands). Epiretinal membranes were send to histologic examination.

### iOCT study

The first commercially available iSD-OCT system, Rescan 700 (Zeiss, Oberkochen, Germany), fully integrated into the surgical microscope (OPMI Lumera 700) was used for intraoperative OCT imaging. The technical characteristics of the iSD-OCT system are following: OCT engine - spectral domain, scanning speed of 27,000 A-scans per second, wavelength of 840 nm, refresh rate - 5 Hz to 50 Hz, axial resolution - 5.5 μm in tissue, scan depth - 2 mm, scan length 6 mm (adjustable from 3 to 16 mm), scan modes - line, 5 lines, and cross-hair. The head-up display injected into the right ocular of the microscope facilitated visual control of iOCT imaging without interruption of the surgery workflow. The surgeon used the foot pedal control to adjust and capture iOCT images. For data documentation, video and snapshots modes were used to image retina and optic disc. For iSD-OCT images analysis and data presentation, a post-processing of the data including 3-dimentional imaging was applied.

### Results

**Fig. 1:**
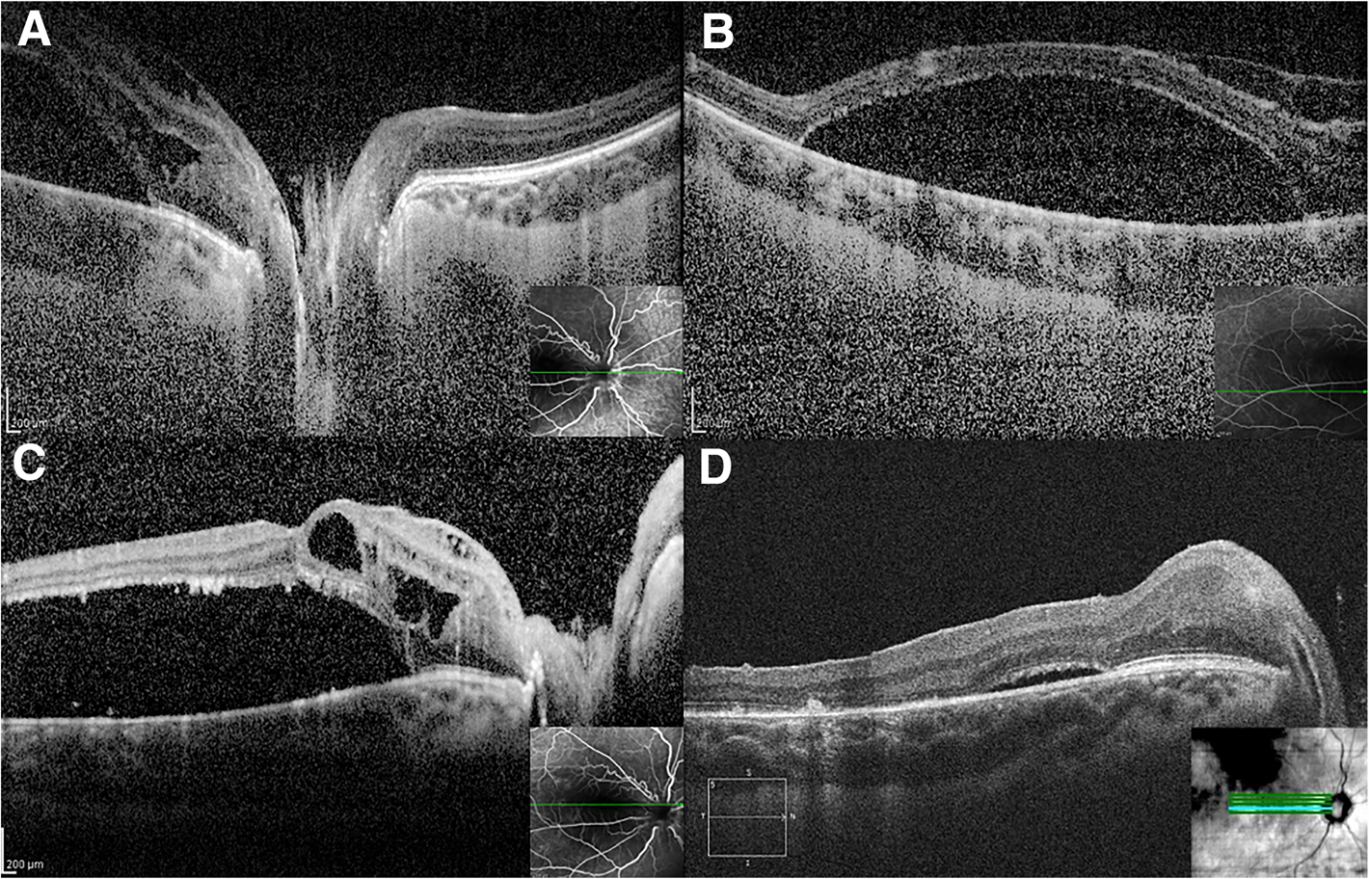
Preoperative OCT if the optic disc (**a**), non-rhegmatogenous RD (**b**) and papillomacular area (**c**). Postoperative OCT of the reattached retina in 4 months follow-up (**d**)


The BCVA on the only right eye dropped from 0.8 to 0.2 (ETDRS chart) during two months’ period before the surgery. Intraocular pressure was 18 mmHg. Pre-operative ophthalmoscopy of the funds of the RE didn’t reveal any retinal break. Preoperative OCT examination of the RE showed retinal detachment of the macular area associated with retinal schisis, epiretinal membrane and cystoid macular changes (Fig. 1b,c). Parapapillary retina was attached (Fig. 1a, Additional file 1: Video S2).

Additional file 1: Video S2. 3D animation of iSD-OCT data of triamcinolone enhanced vitreous attached to the anomalous optic disc in morning glory syndrome. (MP4 22287 kb)


### iOCT of PPV

The condensed vitreous was removed from epipapillary and epimacular area after staining with TA using end-gripping forceps and vitrector (See Additional file 2: Video S1). ERM was removed over the detached retina. ILM was removed after it was stained under the control of iSD-OCT live scanning, in order to make sure that the peeling didn’t affect the thin inner layers of the retinal schisis (See Additional file 2: Video S1). 3-D analysis of OCT images of the optic disc didn’t show any communication between vitreous cavity and subretinal space (Additional file 1: Video S2). There were no central or peripheral retinal breaks found during retina examination. The vitreous cavity was filled only with sterile air and the entry wounds were closed. Patient was positioned face down over the first night after the surgery. Standard postoperative anti-bacterial and anti-inflammatory treatment was prescribed.


Additional file 2: Video S1. iSD-OCT of pars plana vitrectomy for retinal detachment in morning glory syndrome. (MP4 109055 kb)


### iOCT data analysis

#### iSD-OCT imaging of posterior vitreous removal

**Fig. 2:**
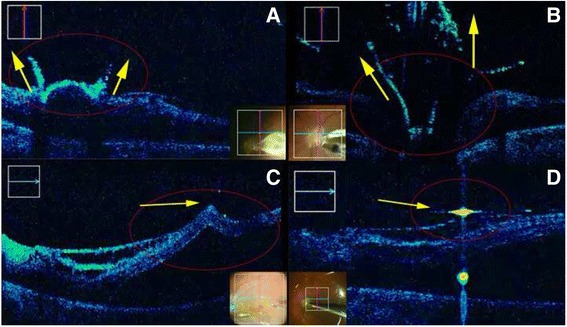
Intraoperative SD-OCT of the triamcinolone enhanced vitreous adhesion in epimacular (**a**) and epipapillary area (**b**). Peeling of the ILM with intact layers of inner retina schisis (**c**). iSD-OCT controlled fluid-air exchange **(d)**

Pars plana vitrectomy necessitated multiple use of triamcinolone to visualise numerous layers of thick vitreous body. Triamcinolone enhanced iSD-OCT imaging showed antero-posterior orientation of strongly adherent hyaloid layers in epimacular (Fig. 2a, yellow arrow) and epipapillary (Fig. 2b, yellow arrow) areas, that proves the presence of vitreoretinal traction (Additional file 1: Videos S2 and Additional file 2: Video S1).

#### iOCT imaging of optic disc and macula

iOCT imaging of the optic disc before and after PPV showed the deep excavation with enlarged dimensions of the disc cup, and epipaillary membranous tissue (Additional file 1: Video S2). Triamcinolone enhanced iOCT assisted for controlled removal of vitreous in the papillomacular and epipapillary areas (Fig. 2b). The vitreous strands were partially left as they were strongly incorporated into anomalous optic disc head (Additional file 1: Videos S2 and Additional file 2: Video S1). iOCT imaging of the optic disc after PPV showed that epipalillary retina remained attached despite the smooth traction caused by vitrectomy. Configuration of the RD after PPV remained unchanged as well. Additionally, iOCT facilitated the visualisation of the tips of the vitrector and end-gripping forceps, helping to avoid the contact between the instruments and the retina and optic disc (Additional file 2: Video S1).

#### iSD-OCT imaging of ERM and ILM removal

ERM was removed after the staining with triamcinolone. The schisis of the inner retinal layers created additional risk for ILM removal. That is why ILM was additionally stained with the dye. iSD-OCT imaging of the macula was used during the initiation of ILM flap and further peeling in order to control the forces of the peeling and approve the unaffected schitic inner retinal layers (Fig. 2c, See Additional file 2: Video S1). Intraretinal cystoid changes remained unchanged after ILM peeling with no evidence of lamellar hole creation, that was documented by iSD-OCT.

Histological study of the ERM was done using the processing in numerous cuts with staining, and it revealed the sparse amorphous periodic acid-Schiff (PAS)-positive matrix without any membranous structure.

#### iSD-OCT imaging during fluid-air exchange

The retina remained detached after fluid-air exchange, as well as retinal schisis remained unchanged. The residual fluid meniscus was detected with iSD-OCT, and it allowed for almost complete fluid-air exchange (Fig. 2d yellow arrow, Additional file 2: Video S1).

In 1 months follow BCVA improved to 0.6 and in 3, 6, 8 and 12 months - to 0.8 (ETDRS chart), respectively. Intraocular pressure remained normal during the whole follow-up period. Postoperative OCT of the macula revealed gradual resolution of intraretinal and subretinal fluid (Fig. 1d). No inflammatory or any other complications in follow-up period were noticed.

## Discussion and conclusions

Pathophysiological causes of exudative retinal detachment with retinal schisis in morning glory disc anomaly have not been clarified. There are many publications of single cases and case series of retinal detachment associated with MGS that describe different therapeutical, surgical and combined treatment approaches [13–27]. The various pathogenesis theories support all types of retinal detachment in MGS: exudative, tractional and rhegmatogenous [13, 19–21, 27].

Bartz-Schmidt and Heimann reported a case, where the retinal hole located within optic disc anomaly supported the connection between vitreous cavity and subretinal space for fluid and heavy liquid to migrate under the retina [19]. Zhang and all reported eight cases of proliferative RD associated with macular hole in young patients with MGS [20]. All patients were successfully treated with PPV, epi- and subretinal membrane removal, endolaser and silicone oil tamponade. Coll and all published a case of RD in 69-year-old patient with MGS with intraoperative evidence of the connection between the vitreous cavity and subretinal space [6].

It has been reported that the most common location for the retinal breaks is in the retinal tissue within anomalous and retracted optic disc (Table 1) [19–24]. Additionally, in majority of cases a long lasting gas or silicon oil tamponades were required (Table 1).

**Table 1:** Analysis of RD and surgical approaches in MGS treatment published previously

Authors	N cases	Age, years	Gender	Type of Retinal Deatchment	Location of the retinal break	Epipapillary membrane	Tratment	Results of the surgery
Bartz-Schmidt and Heimann (1995)	1	6	male	Rhegmatogenous	Within optic disc	Condensed vitreous in epipapillary area	PPV, peripheral retinotomy, endolaser around the hole, silicon oil	Retina reattached, ERM formation in two years
Zhang and all (2013)	8	5–13 (mean 8)		Rhegmatogenous	Abnormal expansion excavation of the optic disc	Epipapillary membrane	PPV, endolaser around the excavation of the optic disc, silicon oil	Retina reattached, silicone oil removed successfully
Ridings and all (1988)	1	14		Rhegmatogenous	Staphylomatouse area		Retinopexy, C3F8 injection	Retina reattached
Akiyama and all (1984)	1	9	female	Rhegmatogenous	Temporal margin of the optic disc		PPV, SRF endodrainage, photocoagulation around the disc	Retina reattached
Yang and Zhang (2010)	1	35	male		Slitlike break near the margin of excavation		PPV, SRF endodrainage, endolaser, 20% C3F8	Retina reattached
Coll and all (1995)	1	69	male	Rhegmatogenous	Slitlike break within optic disc	Epipapillary pigmented membrane	PPV, ERM removal,autologus plasma to seal the hole, silicone oil	Retina reattached, silicone droplets migrated in subretinal space
Ho and Wei (2001)	1		male	Rhegmatogenous	At the edge of excavated disc	Epipapillary fibroglial tissue	PPV, ERM removal, C3F8 tamponade	Retina reattached

In our iSD-OCT analysis of PPV for RD in MGS we revealed a strong adhesion between posterior condensed vitreous and epipapillary area and macula that was seen on iOCT (Fig. 2a,b, Additional file 1: Videos S2 and Additional file 2: Video S1). Based on iSD-OCT data analysis, we assumed that tractional force of the vitreous was the major cause of the retinal detachment in this particular case of MGS, Primary tractional forces of the vitreous can induce tractional RD and further formation of the retinal break at the weakest area of the overstretched retinal tissue within excavated optic disc. In young patients vitreoretinal traction and RD can be caused by enlargement of the axial length, and in older patients - as a result of the natural course of vitreous detachment. We also considered that PPV on early stages of tractional RD in this case prevented retinal break formation and further loss of central VA. Besides that, postoperative retinal reattachment after only air tamponade can also support the fact that vitreoretinal traction caused RD, which was successfully treated after separation of schitic posterior vitreous layers from the retina.

The patients with rhegmatogenous RD due to MGS, that are described in the above mentioned publications, probably presented at later stage of the disease and were operated also [19–24]. Longstanding central retinal schisis potentially can lead to irreversible atrophic changes of the retinal layers due to persistence of intra- and subretinal fluid, and can serve as an additional argument for early PPV.

There was no ERM revealed on histological analysis. ILM folds seen on iOCT proved the tangential traction forces and served as an indication for its removal.

Ehlers and all reported a special surgical technique during PPV for optic disc pit associated RD, during which the surgeon performed active aspiration using the vitrector above the optic disc pit [28]. After the active aspiration retina has reattached intraoperatively, confirming there was a communication between subretinal space and vitreous cavity. In our case of optic disc abnormality, we performed same manoeuvre but didn’t reach any reattachment. In addition, a proper examination of the detached retina using zoom magnification didn’t reveal any retinal break. Furthermore, maculopathy associated with optic disc pit has different origin, which is much more investigated then in MGS cases [29, 30].

There are other reports about different visualisation modality of optic disc anomaly in MGS [31–33]. iSD-OCT in vivo visualisation of vitreo-retinal traction and relations between vitreous and intra- and subretinal space during PPV can assist for better removal of schitic vitreous and control of ERM and ILM peeling, in order to minimise the risks of intrasurgical complications on the diseased eyes.

The limitation of this study is that the iSD-OCT data analysis is based on a single case observation. However, iOCT systems are quite new and expensive technology and are not available in majority of vitreoretinal units. Additionally, RD due to MGS is a rare condition as well, which makes difficult to design and perform a proper study using iSD-OCT.

In MGS there is a strong pathologic adhesion between the vitreous and central cetinal. Pathologic changes of the vitreous with further traction and contraction can lead to the RD.

The surgery on the only eye with MGS can be difficult and risky. iSD-OCT assisted PPV in RD case due to MGS on the only eye facilitated to remove thick layers of the vitreous and separate ILM from schitic retina much safer. The longstanding vireo-retinal traction and intra- or subretinal fluid can lead to retinal break formation and retinal atrophy, respectively. PPV has to be considered on the early stages of RD due to MGS.

## Abbreviations

BCVA: Best corrected visual acuity
ERM: Epiretinal membrane
ILM: Internal retinal membrane
iSD-OCT: Intraoperative spectral domain optical coherent tomography
MGS: Morning glory syndrome
Non-RRD: Non-rhegmatogenous retinal detachment
OCT: Optical coherence tomography
PPV: Pars plana vitrectomy
RD: Retinal detachment
RE: Right eye
TA: Triamcinolone acetonide
